# Pancreatic Perivascular Epithelioid Cell Tumor (PEComa) Mimicking Neuroendocrine Tumor (NET): A Case Report

**DOI:** 10.7759/cureus.94674

**Published:** 2025-10-15

**Authors:** Shao-En Hung, Yu-Ting Yu

**Affiliations:** 1 Department of Emergency Medicine, Jen-Ai Hospital, Taichung, TWN; 2 Department of Emergency Medicine, Chung Shan Medical University Hospital, Taichung, TWN; 3 Department of Emergency Medicine, Taichung Hospital, Ministry of Health and Welfare, Taichung, TWN; 4 Department of Pathology, Chung Shan Medical University, Taichung City, TWN; 5 Department of Pathology, Chung Shan Medical University Hospital, Taichung City, TWN; 6 Department of Pathology, Taipei City Hospital Heping Fuyou Branch, Taipei, TWN

**Keywords:** central pancreatectomy, neuroendocrine tumor, pancreatic pecoma, who classification, perivascular epithelioid cell tumor (pecoma)

## Abstract

Pancreatic perivascular epithelioid cell tumors (PEComas) are exceedingly rare mesenchymal neoplasms that often mimic neuroendocrine tumors (NETs) in their clinical and radiologic presentation, making preoperative diagnosis challenging. We report the case of a 67-year-old female with a 1.7 cm pancreatic body tumor initially suspected to be a NET. Preoperative chromogranin A levels were within the normal range. The patient underwent central pancreatectomy, and histopathological examination revealed a neoplasm composed of epithelioid cells arranged in sheets and cords within a hyalinized stroma. The tumor cells exhibited vesicular nuclei with distinct nucleoli and eosinophilic to clear cytoplasm. Although nuclear pleomorphism was present, no increased mitotic activity or tumor necrosis was identified. Immunohistochemistry was positive for HMB-45 and Melan-A, confirming the diagnosis of pancreatic PEComa. This report underscores the diagnostic challenge of pancreatic PEComas, which can closely resemble NETs, and highlights the importance of immunohistochemistry in establishing a definitive diagnosis.

## Introduction

Perivascular epithelioid cell tumors (PEComas) refer to a rare and distinctive family of mesenchymal tumors, defined by their unique morphology and immunophenotype [1,2]. These neoplasms are composed of epithelioid cells with clear to eosinophilic cytoplasm, and they characteristically co-express melanocytic markers (like HMB-45, Melan-A, and MITF) and smooth muscle markers (such as smooth muscle actin (SMA) [1-5]. The term PEComa, first introduced in 1994 [3], refers to a family of tumors characterized by perivascular epithelioid cells, including angiomyolipoma, lymphangioleiomyomatosis, and clear cell “sugar” tumor of the lung. These tumors can arise in various anatomical sites, with the uterus and kidneys being the most frequently reported [4]. However, their occurrence in the pancreas is exceptionally rare, with only a handful of cases documented in the medical literature so far [2,5,6]. 

The rarity of pancreatic PEComas, combined with their non-specific clinical and radiological features, presents a significant diagnostic challenge [2,5,6]. Patients may be asymptomatic, with the tumor discovered incidentally during imaging for other reasons, or they may present with symptoms related to the tumor's mass effect, such as abdominal pain or jaundice. On imaging studies such as CT and MRI, pancreatic PEComas often show non-specific characteristics that overlap with other more common pancreatic neoplasms [7-10]. This makes it particularly difficult to differentiate them from pancreatic neuroendocrine tumors (PNETs), which are also well-vascularized and can present similarly. The clinical and radiological mimicry between PEComas and PNETs can lead to misdiagnosis, highlighting the necessity of a definitive pathological diagnosis.

Given the diagnostic ambiguity, the definitive diagnosis of a pancreatic PEComa relies on a combination of histological examination and immunohistochemical staining. Histologically, these tumors show a unique perivascular growth pattern and a cellular composition of epithelioid cells with clear to eosinophilic cytoplasm [1,4]. However, immunohistochemistry is crucial for confirmation. The co-expression of melanocytic markers, particularly HMB-45, is a hallmark of PEComas and is essential for distinguishing them from other tumors [4], especially PNETs, which are positive for neuroendocrine markers like chromogranin A and synaptophysin.

Recent studies have highlighted the role of TSC1/TSC2 gene mutations and mTOR pathway activation in PEComas, which not only contribute to tumorigenesis but also provide potential therapeutic targets. mTOR inhibitors have shown efficacy in select patients with advanced or unresectable PEComas, underscoring the translational relevance of these molecular insights. Updated World Health Organization (WHO) classification criteria for malignancy mention tumor size, mitotic activity, and necrosis [1,10]. This case report details a rare instance of a pancreatic PEComa in a 67-year-old female that was initially suspected to be a neuroendocrine tumor based on imaging.

## Case presentation

**Figure 1:**
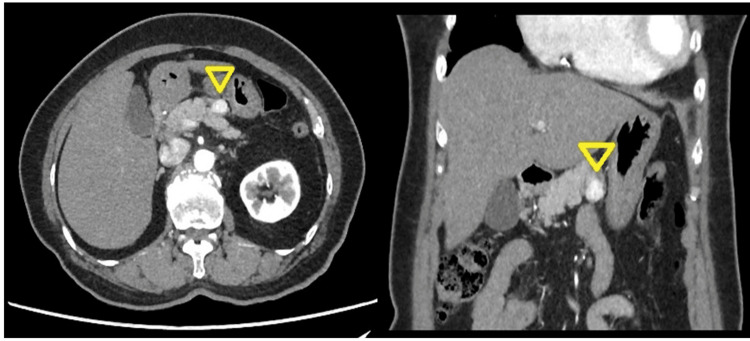
CT image of the tumor (A) Axial view of contrast-enhanced CT reveals a hypervascular lesion (arrowhead) situated in the body of the pancreas. (B) Coronal view of contrast-enhanced CT demonstrates the extent of the pancreatic tumor (arrowhead) CT: computed tomography

A 67-year-old female presented to the department of gastroenterology at our hospital with a one-month history of diffuse, dull abdominal pain. The pain was continuous, worse when leaning forward, but did not radiate to other regions and did not significantly interfere with her daily activities. She had no associated symptoms such as nausea, vomiting, fever, or weight loss. Laboratory investigations, including tumor markers, showed that her serum chromogranin A level was within the normal range, and all other routine blood tests were unremarkable. A CT scan revealed a well-circumscribed, hypervascular 1.7-cm mass located in the body of the pancreas (Figure 1), initially suspected to be a pancreatic NET based on its imaging characteristics. The patient had no significant past medical history.

Given the suspicion of a pancreatic neoplasm, and after thorough discussion with the patient, a laparoscopic central pancreatectomy was performed. Intraoperatively, the tumor was well-demarcated and confined to the pancreatic body, and the specimen was excised with an approximately 1-cm margin of surrounding pancreatic tissue.

**Figure 2:**
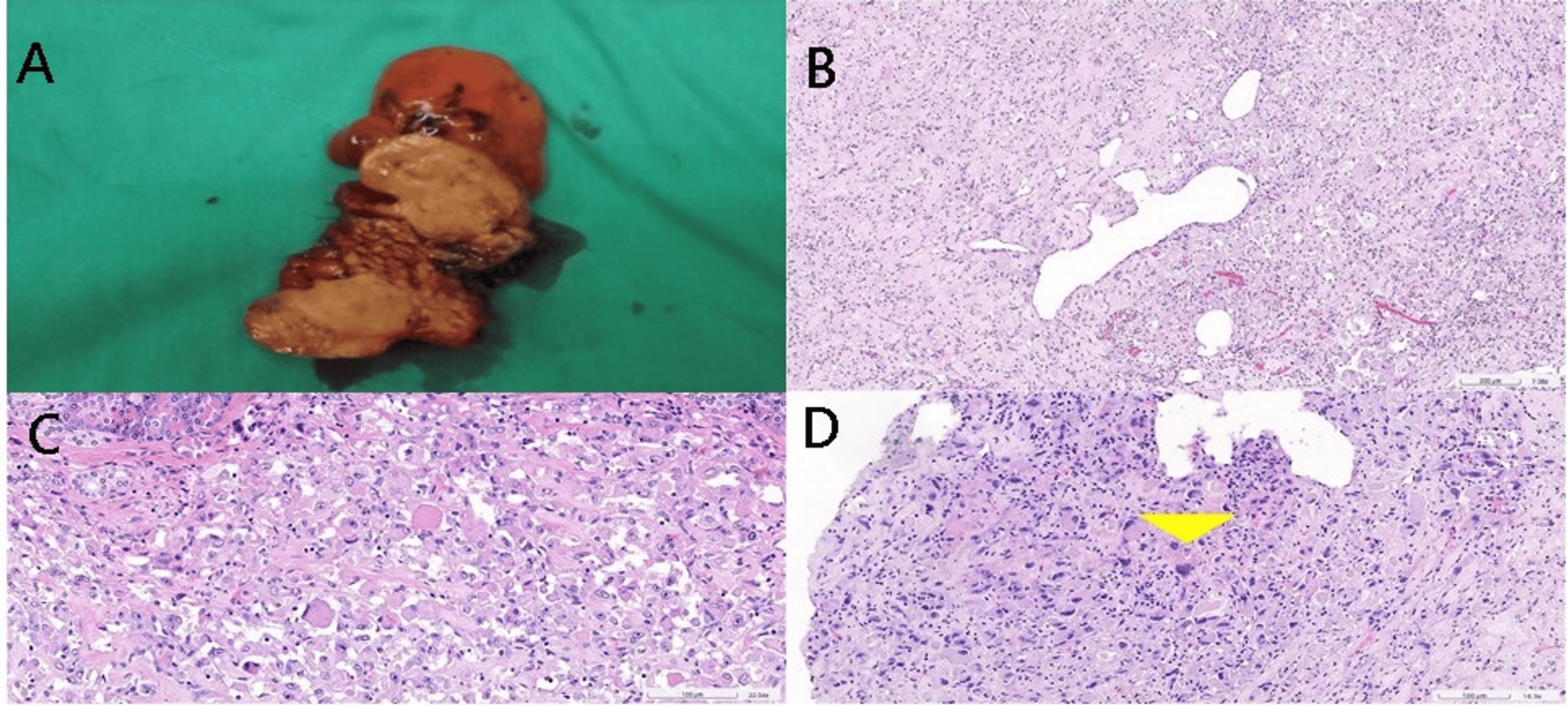
Gross and microscopic findings from the specimen (A) Gross examination reveals a well-circumscribed tumor with a tan-colored cut surface. (B) Microscopic examination shows that the tumor is composed of epithelioid cells arranged in sheets, cords, and nests. The tumor cells are closely associated with blood vessels. (C) The tumor has abundant eosinophilic cytoplasm (D) Focally, multinucleated giant cells (arrowhead) and nuclear pleomorphism are observed

Gross examination of the resected specimen revealed a well-circumscribed, solid, and homogenous mass measuring 1.7 cm in its greatest dimension (Figure 2A). Low-power microscopic examination confirmed the well-circumscribed nature of the tumor. Histologically, the tumor was composed of epithelioid neoplastic cells arranged in sheets, cords, or vague nests within a prominent hyalinized stroma, with a close association to blood vessel walls (Figure 2B). These neoplastic cells exhibited vesicular nuclei with distinct nucleoli and abundant eosinophilic or clear cytoplasm (Figure 2C). Multinucleated giant cells and nuclear pleomorphism were also observed within the tumor (Figure 2D). Also, no increased mitotic activity (<1 mitosis per 50 high-power fields) or tumor necrosis was identified.

Immunohistochemical staining was performed, demonstrating strong positivity for HMB45 and Melan-A, and focal positivity for SMA. The tumor cells were negative for cytokeratin, synaptophysin, and chromogranin, ruling out NET and confirming the diagnosis of pancreatic PEComa. Based on these characteristic histopathological features and the distinctive immunohistochemical profile, a final diagnosis of pancreatic PEComa was made. The patient's postoperative course was uneventful, and she remained asymptomatic at the six-month follow-up.

## Discussion

The WHO classification of soft tissue and bone tumors provides guidelines for the diagnosis and prognostication of PEComas. While most PEComas behave benignly, some exhibit malignant potential. The 2020 WHO classification builds upon previous criteria to stratify PEComas into categories of "benign," "uncertain malignant potential," and "malignant." Several histopathologic features are considered predictive of malignant behavior, including a tumor size of 5 cm or greater, an infiltrative growth pattern, high nuclear grade or atypia, increased mitotic activity, tumor necrosis, and vascular invasion [1].

Pancreatic PEComas are extremely rare mesenchymal tumors, representing a diagnostic challenge due to their variable clinical presentations and radiological mimicry of other more common pancreatic neoplasms [2]. The vast majority of PEComas are benign, while a subset can exhibit malignant behavior [1]. Our patient's case further underscores the difficulty in preoperative diagnosis, as the tumor was initially considered a NET, a more frequent pancreatic entity, based on its imaging appearance. The normal preoperative chromogranin level, while not definitively excluding a NET, contributed to the diagnostic uncertainty. Definitive diagnosis invariably relies on histopathological examination and a comprehensive immunohistochemical panel.

Pancreatic PEComas tend to affect middle-aged to elderly individuals, with a slight female predominance [4-6]. Clinically, they often present with non-specific abdominal symptoms or are discovered incidentally [2,7,8]. Radiologically, pancreatic PEComas often appear as well-circumscribed masses, which can be hypervascular or show variable enhancement patterns. This imaging characteristic can overlap with NETs, which are also often hypervascular. Therefore, imaging alone is insufficient for establishing a definitive diagnosis [2,9].

Pathologically, PEComas are characterized by epithelioid or spindle cells arranged around blood vessels. The cells typically have clear or eosinophilic granular cytoplasm. The hallmark of PEComa diagnosis is the co-expression of melanocytic markers (such as HMB45, Melan-A) and smooth muscle markers (such as SMA, desmin). The absence of synaptophysin and chromogranin staining in our case was crucial in distinguishing the tumor from a NET [10]. The presence of multinucleated giant cells and nuclear pleomorphism, as observed in our patient, can be seen in PEComas and does not automatically indicate malignancy in the absence of other high-risk features.

In our case, while nuclear pleomorphism and multinucleated giant cells were present, the absence of increased mitotic activity, tumor necrosis, infiltrative growth, and the relatively small tumor size (1.7 cm) suggested a low likelihood of aggressive behavior. Thus, this pancreatic PEComa would likely be classified as having benign or uncertain malignant potential based on these criteria. However, it is important to acknowledge that our patient has had only six months of follow-up, which is insufficient to draw definitive conclusions regarding long-term prognosis. Given the unpredictable biological behavior of PEComas, ongoing long-term surveillance remains essential, regardless of initial risk stratification [11,12].

## Conclusions

Pancreatic PEComas are rare tumors that pose a significant diagnostic challenge, particularly because they can mimic more common pancreatic neoplasms such as NETs. Accurate diagnosis relies on histopathological examination combined with a comprehensive immunohistochemical panel. Awareness of PEComas, their characteristic pathological features, and the application of the updated WHO malignancy criteria are crucial for pathologists and clinicians to ensure appropriate management and follow-up.
